# A vision for closing the evidence-practice gap in the management of low-grade prostate cancer

**DOI:** 10.1093/jncics/pkad028

**Published:** 2023-04-24

**Authors:** Michael S Leapman, Stacy Loeb, Matthew R Cooperberg, William J Catalona, Franklin D Gaylis

**Affiliations:** Department of Urology, Yale School of Medicine, New Haven, CT, USA; Yale Cancer Outcomes, Public Policy, and Effectiveness Research Center, New Haven, CT, USA; Department of Chronic Disease Epidemiology, Yale School of Public Health, New Haven, CT, USA; Departments of Urology and Population Health, New York University Langone Health; Manhattan Veterans Affairs Medical Center, New York, NY, USA; Department of Urology, University of California San Francisco, San Francisco, CA, USA; Department of Urology, Northwestern University Feinberg School of Medicine, Chicago, IL, USA; Genesis Healthcare Partners, San Diego, CA, USA

Gaps between clinical evidence and practice plague all domains of medicine. The time needed to translate the highest-quality evidence into clinical care is commonly estimated to be 17 years, an almost inconceivably slow pace (1,2). In this issue of the Journal, Borregales and colleagues (3) show that the conservative management of low-grade (ie, Gleason grade group 1) prostate cancer is no exception. Pathbreaking work over decades has chartered an evidentiary course supporting initial active surveillance as the preferred strategy for most men with low-grade prostate cancer (4,5). This approach has stood on solid ground for more than a decade and, in many instances, defers or avoids the possible adverse consequences of immediate treatment (6). Among patients with low-risk features—typically identified by the cancer grade, stage, and prostate-specific antigen (PSA) level—active surveillance is supported by level 1 evidence, a trove of high-quality prospective clinical and biological studies, and national guidelines. However, left to passive diffusion alone, active surveillance has been sluggish in uptake, highly varied in the quality of delivery, and often subject to premature termination (7).

Borregales et al. (3) present data from the National Cancer Database, a hospital registry drawn from Commission on Cancer–accredited facilities, showing that as of 2019, conservative management (active surveillance, watchful waiting, or no immediate treatment) has eclipsed definitive treatment for low-risk prostate cancer in academic medical centers (62%) and is poised to do so in community-affiliated sites (49%). Notably, the landmark work on the feasibility of active surveillance appeared in 2002, 17 years before these data were collected (4). These findings align with earlier reports from other cross-sectional and population-based sources and offer encouragement that overtreatment of low-grade prostate cancer is receding but far too slowly (8,9). Moreover, the quality of surveillance in the United States varies dramatically by physician, practice, and region (10). Clinicians frequently omit critical aspects of surveillance, such as a confirmatory prostate biopsy, and patients frequently transition to active treatment without justifiable cause (11).

The study by Borregales et al. (3) and other recently published data suggest the United States is finally reaching an inflection point in the initial uptake of active surveillance; however, it would be unwise to view modest gains as sufficient or inevitable (12). There is an urgent need to redouble the collective efforts to accelerate the adoption of high-quality active surveillance for low-risk prostate cancer. A multitiered strategy is necessary to overcome the many obstacles encountered. The core components should include 1) further scientific advancements to refine the specificity and practice of active surveillance, 2) leveraging proven principles of behavior change (implementation science) to optimize patient and physician decision choice and management quality, and 3) alignment of economic incentives for delivering high-quality, evidence-based practice (value-based care) by payors committed to improving population health.

Accurate and sufficiently detailed clinical data are the bedrock of any plan to improve health-care quality in the modern era. Reliable information is required to define clinical needs, set benchmarks, calibrate clinical models, and track progress. Borregales et al. (3) highlight the limitations of existing national datasets. The National Cancer Database and other claims-based resources such as the Surveillance, Epidemiology and End Results–Medicare linkage offer cross-sectional US cancer diagnosis and treatment trends data but lack granularity regarding the cancer characteristics, human motivations, or the nature of observational monitoring. Thus, investigators cannot distinguish between fundamentally different strategies (eg, active surveillance vs watchful waiting) or account for the important subtleties in cancer volume or distribution that may drive decision making. Leveraging electronic health record resources, via broad efforts such as those reflected in the American Urological Association Quality registry, will allow investigators to shine a more focused light on trends and determinants of patient outcomes (12).

A new vision for localized prostate cancer also must account for the changing face of the disease. Adoption of active surveillance is increasing among patients with Gleason grade group 2 disease and carries nontrivial risks of metastatic progression. The prospect of stage migration warrants acute attention, as highlighted by the findings of Borregales et al. (3) that low-grade (grade group 1) prostate cancer is no longer the most commonly diagnosed grade in the United States. Detection of higher-grade disease could be due to greater use of prebiopsy risk tools and more nuanced decision making; however, we may also be observing the late rippling effects of less PSA-based screening that was set into motion by the US Preventive Services Task Force grade D recommendation against PSA screening in all men in 2012 (13). Refined biopsy techniques aided by prostate magnetic resonance imaging also may have improved the identification of occult, higher-grade disease previously missed on systematic biopsies alone, reclassifying some patients previously classified as low-grade. Thus, there is a pressing need to expand the study of active surveillance to patients with grade group 2 disease.

To bridge the divide between clinical guidelines and clinical practice, physicians need the necessary tools for evidence-based care for low-grade prostate cancer. As shown by Borregales et al. (3) and lessons from the past 2 decades, a laissez-faire approach toward managing these patients will continue to yield highly fragmented results and lead to overtreatment. Numerous empiric successes in changing practices have been achieved by engaging the principles of implementation science, such as incentivization and performance feedback (13,14). To ensure success, physicians cannot go it alone. Patient advocates and payors must remain prominent partners in this goal as medicine shifts toward reimbursement models prioritizing health-care quality over quantity (15).

## Data Availability

No new data were generated or analyzed for this editorial.
